# Bacterial‐Mediated Tumor Therapy: Old Treatment in a New Context

**DOI:** 10.1002/advs.202302957

**Published:** 2023-06-23

**Authors:** Yao Liu, Lili Niu, Nannan Li, Yang Wang, Mingyang Liu, Xiaomin Su, Xuhui Bao, Bo Yin, Shun Shen

*Adv. Sci*. **2022**, 9, 2205641

DOI: 10.1002/advs.202205641

In the original published article, Bo Yin's affiliation is wrong. Please find below the correct affiliations.

Y. Liu

Key Laboratory of Spine and Spinal Cord Injury Repair

and Regeneration of Ministry of Education

Orthopaedic Department of Tongji Hospital, The Institute for Biomedical

Engineering and Nano Science

Tongji University School of Medicine

Shanghai 200092, P. R. China

Y. Liu, S. Shen

Pharmacy Department and Center for Medical Research and Innovation

Shanghai Pudong Hospital

Fudan University Pudong Medical Center

Shanghai 201399, China

E‐mail: sshen@fudan.edu.cn

L. Niu, N. Li, Y. Wang, X. Su

Central Laboratory

First Affiliated Hospital

Institute (College) of Integrative Medicine

Dalian Medical University

Dalian 116021, China

M. Liu

Department of Surgical Oncology and General Surgery

The First Hospital of China Medical University

155 North Nanjing Street, Heping District, Shenyang 110001, China

X. Bao

Institute for Therapeutic Cancer Vaccines and Department of Oncology

Fudan University

Pudong Medical Center

Shanghai 201399, China

E‐mail: xuhui.bao@duke.edu

B. Yin

Department of Radiology

Huashan Hospital

Fudan University

Shanghai 200040, China

E‐mail: yinbo@fudan.edu.cn

